# Supplementary material for: Generalized entropies, density of states, and non-extensivity
Source: Sci Rep. 2020 Sep 23;10:15516. doi: 10.1038/s41598-020-72422-8 (PMC7511985; doi:10.1038/s41598-020-72422-8)
Supplement: Supplementary file 1 — Supplementary Information [file 41598_2020_72422_MOESM1_ESM.pdf]

Supplementary Information - Generalized entropies, density of states, and non-extensivity

Sámuel G. Balogh[1,*], Gergely Palla[2], Péter Pollner[2], and Dániel Czégel[3,4,5,+]

[1]Dept. of Biological Physics, Eötvös University, H-1117 Budapest, Hungary
[2]MTA-ELTE Statistical and Biological Physics Research Group, Dept. of Biological Physics, Eötvös University, H-1117 Budapest, Hungary
[3]Institute of Evolution, Centre for Ecological Research, H-8237 Tihany, Hungary
[4]Dept. of Plant Systematics, Ecology and Theoretical Biology, Eötvös University, H-1117 Budapest, Hungary
[5]Center for the Conceptual Foundations of Science, Parmenides Foundation, 82049 Pullach/Munich, Germany
[*]balogh@hal.elte.hu
[+]danielczegel@gmail.com

Generalized entropies and Jensen inequality

Generalized entropies defined by Eq. (3) are strictly positive $S_g \geq 0\ \forall\{g(p),\rho(p)\}$ since $g(p),\rho(p) \geq 0\ \forall p \in [0,1]$. Besides that, they satisfy the second Shannon-Khinchin axiom (SK2), namely that systems given in their most disordered states are characterized by equiprobable-distributions (entropy is maximal for the uniform distribution). By imposing concavity upon $g(p)$, this can easily be verified through the well-known Jensen's inequality (J.I.) for concave functions:

$$S_g = W\left\langle g(p)\right\rangle_\rho \overset{\text{J.I.}}{\leq} Wg\left(\langle p\rangle_\rho\right) = Wg\left(\frac{1}{W}\right) = W\left\langle g(p)\right\rangle_{\rho=\delta(p-1/W)} = \max_\rho S_g, \tag{S1}$$

where we have exploited both normalization and expected value constraints.

Density of states: examples

Uniform distribution over configuration space (microcanonical)

Despite its triviality the microcanonical ensemble is unambiguously among the most relevant examples since it is an essential part of thermodynamics, statistical physics as well as information theory.

It is defined by uniform probabilities over the configuration space, that is $\rho = \delta(p - 1/W)$, whose normalization can simply be deduced from the definition of Dirac delta function. Correspondingly, expected value condition is given by

$$\langle p\rangle_\rho = \int_0^1 p \cdot \delta\left(p - \frac{1}{W}\right)\mathrm{d}p = \frac{1}{W} \tag{S2}$$

in agreement with Eq. (4).

Multiple uniform domains over configuration space (multi-delta)

Multi-delta is the generalization of the microcanonical picture, where different sets of configurations are given. Within these sets each configuration are equiprobable however, configuration belonging to different sets might have different probabilites assigned to them.

The corresponding $\rho(p)$ defined by Eq. (9) is normalized since

$$\int \rho(p)\mathrm{d}p = \frac{V_0}{W} + \sum_{j=1}^{k}\frac{V_j}{W} = \sum_{j=0}^{k}\frac{V_j}{W} = 1, \tag{S3}$$

whereas expected value constraint can be verified by evaluating

$$\langle p\rangle_\rho = \int p\rho(p)\mathrm{d}p = \frac{V_0}{W}\cdot 0 + \sum_{j=1}^{k}\frac{V_j}{W}\frac{V_j}{\sum\limits_{m=1}^{k} V_m^2} = \frac{1}{W}. \tag{S4}$$

Once normalization and expected value conditions are imposed, one can easily check that under the multiplicative rescaling of confiuration space volume BGS entropy scales as

$$R_\lambda = \lim_{W\to\infty} \frac{V^*(\lambda W) g_{\mathrm{BGS}}\left(\frac{1}{V^*(\lambda W)}\right)}{V^*(W) g_{\mathrm{BGS}}\left(\frac{1}{V^*(W)}\right)} = \lim_{W\to\infty} \lambda^{\xi} \frac{\frac{1}{(\lambda W)^{\xi}} \ln \frac{1}{(\lambda W)^{\xi}}}{\frac{1}{W^{\xi}} \ln \frac{1}{W^{\xi}}} \sim \text{const.}, \tag{S5}$$

implying that $c = 1$ in accordance with the corresponding row in Table1. Consequently, multi-delta density of states can not change the c exponent, therefore, BGS entropy remains extensive under $W(N) \sim e^N$.

Considering the case of Tsallis entropy, we take the $p \to 0^+$ limit (for $\xi > 0$) and utilize the expansion of $g_q(p)$ around $p = 0$

$$g_q(p) = \begin{cases} \frac{1}{1-q} p^q + \mathscr{O}(p) & \text{if } 0 < q < 1 \\ \\ -\frac{1}{1-q} p + \mathscr{O}(p) & \text{if } q > 1, \end{cases} \tag{S6}$$

based on which we obtain

$$R_\lambda \approx \frac{V^*(\lambda W)\left(\frac{1}{V^*(\lambda W)}\right)^q}{V^*(W)\left(\frac{1}{V^*(W)}\right)^q} \tag{S7}$$

for $0 < q < 1$. Assuming the scaling form of $V^*(W) \sim W^{\xi}$ the previous equation can be further simplified to

$$R_\lambda = \frac{(\lambda W)^{\xi}\left(\frac{1}{(\lambda W)^{\xi}}\right)^q}{W^{\xi}\left(\frac{1}{W^{\xi}}\right)^q} \sim \lambda^{1-c}, \tag{S8}$$

where $c = 1 - \xi + \xi q$ in agreement with the corresponding row in Table2.

A single macroscopic state (Bose-Einstein)

Owing to the presence of a macroscopic state, H-T scaling and thus, conditions of extensivity substantially change under Bose-Einstein density of states. This stems from the fact that states with probabilites $p \approx 1$ have non-negligible contribution to the entropy compared to, e.g. the microcanonical picture where the major contribution is coming from $p = \frac{1}{W} \approx 0$.

The corresponding density of states written in the form of Eq. (14) fulfills both normalization and expected value contraints since the following identities hold

$$\int \rho(p)\mathrm{d}p = \frac{1}{W} + \frac{W-1}{W} = 1 \quad \text{and} \quad \langle p \rangle_\rho = \frac{1}{W}\left(1 - \frac{1}{W-1}\right) + \frac{W-1}{W}\frac{1}{(W-1)^2} = \frac{1}{W}. \tag{S9}$$

Plugging Eq. (14) into Eq. (3) and exploiting the fact that

$$\int f(x)\delta(x - x_0)\mathrm{d}x = f(x_0) \tag{S10}$$

with $\delta(x)$ being the Dirac-delta function, we simply arrive to

$$S_g = g\left(1 - \frac{1}{W-1}\right) + (W-1)g\left(\frac{1}{(W-1)^2}\right). \tag{S11}$$

Specifically, for the BGS entropy we obtain

$$S_{\mathrm{BGS}} = \frac{W-2}{W-1}\ln\left(\frac{W-1}{W-2}\right) + \frac{2}{W-1}\ln(W-1). \tag{S12}$$

With the notations of $x = \frac{1}{W-1}$ and $z = \frac{1}{\lambda}$ the generalized c exponent for BGS entropy is given by directly plugging Eq. (S11) into Eq. (5) yielding

$$\begin{aligned} R_z = \lim_{x\to 0^+} \frac{g_{\mathrm{BGS}}(1 - zx) + \frac{1}{zx} g_{\mathrm{BGS}}\left((zx)^2\right)}{g_{\mathrm{BGS}}(1-x) + \frac{1}{x} g_{\mathrm{BGS}}(x^2)} &= \lim_{x\to 0^+} \frac{(1-zx)\ln(1-zx) + zx\ln\left((zx)^2\right)}{(1-x)\ln(1-x) + x\ln(x^2)} \\ \approx \frac{z - 2z\ln(zx)}{1 - 2\ln(x)} \sim z \equiv z^{c-1}, \end{aligned} \tag{S13}$$

where $c = 2$. Interestingly, extensivity of BGS can be maintained by imposing $W(N) \sim e^{-\mathscr{W}_{-1}\left(-\frac{N}{2}\right)}$. The previous form of $W(N)$ is a decreasing function of N implying that asymptotically, $N \to \infty$, the configuration space has to collapse in order for extensivity to be satisfied.

Under Bose-Einstein density of states Tsallis entropy reads

$$S_q = \frac{1 - \left(1 - \frac{1}{W-1}\right)^q - \left(\frac{1}{W-1}\right)^{2q-1}}{q-1}. \tag{S14}$$

Based on Eq. (S14) and Eq. (S6) generealized H-T scaling takes the form of

$$R_z \approx \lim_{x \to 0^+} \frac{zx - (zx)^{2q-1}}{x - x^{2q-1}} \sim z^{2q-1} \equiv z^{c-1}, \tag{S15}$$

where $c = 2q$. For $q = \frac{1}{2}$, $S_q \to 1$ therefore Tsallis entropy can not be made extensive. In case of $q \in \left(0, \frac{1}{2}\right)$ extensivity is obtained by setting $W(N) \sim N^{\frac{1}{1-2q}}$. If however, $q > \frac{1}{2}$ $S_q \sim N$ requires $W(N)$ to decrease with N.

Exponential density of states (exponential)

Under exponential form of ρ noramlization condition gives

$$\int_0^1 W e^{-Wp} \mathrm{d}p = 1 - e^{-W} \approx 1, \tag{S16}$$

while its expected value is simply written as

$$\langle p \rangle_\rho = \int_0^1 pWe^{-Wp} \mathrm{d}p = \frac{1 - e^{-W}(W+1)}{W} \approx \frac{1}{W}, \tag{S17}$$

where the approximations are obtained by neglecting asymptotically vanishing terms. Under this form of ρ BGS entropy is given by

$$S_{\mathrm{BGS}} = \ln W + \mathrm{Shi}(W) - \mathrm{Chi}(W) + e^{-W} - 1 + \gamma \tag{S18}$$

where γ is the Euler-Mascheroni constant while $\mathrm{Shi}(x)$ and $\mathrm{Chi}(x)$ denote the hyperbolic sine and cosine integrals respectively. In the asymptotic limit of $W \to \infty$, Eq. (S18) reduces to the regular $S_{\mathrm{BGS}} \approx \ln W$ form.

Moreover, Tsallis entropy with deformation parameter q takes the form of

$$S_q = W \left\langle \frac{p - p^q}{q-1} \right\rangle_\rho = W^{-(q-1)} \frac{\Gamma(q+1, W) - \Gamma(q+1)}{q-1} + \frac{1 - e^{-W}(W+1)}{q-1}, \tag{S19}$$

with $\Gamma(x,y)$ and $\Gamma(x)$ denoting the Incomplete gamma and Gamma functions respectively. The previous equality along with the asymptotic expressions of $\Gamma(q+1, W \to \infty) = 0$ and $\lim_{W \to \infty} \frac{e^{-W}(W+1)}{q-1} = 0$ suggest

$$S_{q<1} \overset{W \to \infty}{=} \frac{1 - \Gamma(q+1) W^{1-q}}{q-1}. \tag{S20}$$

Exponential distribution represents a particularly fast form of decay implying that the major contribution to entropies is coming from configurations where $p \approx W^{-1}$. This is supported by the fact that the corresponding green curves in Figure 2b are quite close to the Heaviside step function (realizing the microcanonical ensemble). As a consequence, requirements for extensivity do not change and the H-T exponents given by Eq. (5) happen to have the same values as in case of microcanonical ensemble (for details see Table1 and Table2).

Log-gamma density of states (log-gamma, limiting log-normal)

The appropriate parametrization of the log-gamma density of states is being carried out by setting up the following definite integral

$$I_{\mathrm{Log}}(m, w) = \int_0^1 p^m \frac{\ln^w\left(\frac{1}{p}\right)}{\Gamma(w+1)} \mathrm{d}p = (m+1)^{-(w+1)}, \tag{S21}$$

based on which the noramlization condition can automatically be verified by plugging $m=0$ into Eq. (S21). Note that if however, $m=1$ we obtain $\langle p\rangle = 2^{-w-1}$ which results in fulfilling expected value constraint only in case of $w=\log_2(W)-1$.

Boltzmann-Gibbs-Shannon entropy is simply related to the evaluation of a similar integral, namely

$$S_{\rm BGS} = W\int_0^1 p\frac{\ln^{w+1}\left(\frac{1}{p}\right)}{\Gamma(w+1)}\mathrm{d}p = W(w+1)\int_0^1 p\frac{\ln^{w+1}\left(\frac{1}{p}\right)}{\Gamma(w+2)}\mathrm{d}p = \frac{W(w+1)}{2^{w+2}}. \tag{S22}$$

Using the fact that $w=\log_2(W)-1$ Shannon entropy given in its simplest form

$$S_{\rm BGS} = W\frac{\log_2(W)}{2W} \sim \ln W. \tag{S23}$$

Note that expected value constraint generally requires the precise evaluation of $I_{\rm Log}(1,w)$ while Tsallis entropy can be expressed as

$$S_q = W\frac{I_{\rm Log}(1,w)-I_{\rm Log}(q,w)}{q-1} = \frac{1-W(q+1)^{-\log_2(W)}}{q-1}, \tag{S24}$$

which altogether with the identity of $1+q=2^{\log_2(1+q)}$ yield to

$$S_q = \frac{1-W^{1-\log_2(1+q)}}{q-1} \equiv \frac{1-W^{1-c}}{q-1}, \tag{S25}$$

where $c=\log_2(1+q)$ with the corresponding curve depicted in Figure 2c. As a surprising consequence, extensivity of the Tsallis entropy requires $W(N)\sim N^{\frac{1}{\log_2(1+q)}}$.

Let us proceed with the detailed description of the spin model introduced in the main text whose underlying density of states is jointly related to the log-gamma and multi-delta density of states. The ρ_γ associated with this system can be written as a sum, namely

$$\rho_\gamma(p) = \rho_1(p)+\rho_2(p) \tag{S26}$$

with the first and second term accounting for the forbidden and allowed configurations, respectively. Since the effective size of the configuration space is given by V^*, out of $W=2^N$ configurations $W-V^*$ have zero probability to occur, therefore $\rho_1(p)=\frac{W-V^*}{W}$. The second term in Eq. (S26) is bit more involved to evaluate. For the sake of detailed description let us suppose that the probabilities of the sub-domains for strictly pointing upward and downward are denoted by r_j and $1-r_j:\ j=0,\ldots,N_{\rm eff}$, respectively. According to the definition of the model, these r values are drawn from a uniform distribution defined on $[0,1]$. Consequently, the probability of the k-th spin configuration can be given by a product of random variables, $R_k=\prod_{j=1}^{N_{\rm eff}} r_j^{\theta_j(k)}(1-r_j)^{1-\theta_j(k)}$, $k=0,\ldots,2^{N_{\rm eff}}$, where $\theta_j(k)=1$ if the j-th spin points upward and $\theta_j=0$ if it points downward in the k-th configuration. With the above arguments Eq. (S26) yields to

$$\rho_\gamma(p) = \frac{W-V^*}{W}\delta(p)+\eta\sum_{k=1}^{2^{N_{\rm eff}}}R_k, \tag{S27}$$

where η is a multiplicative factor which guarantees normalization. Note that if r_i-s are uniformly and independently distributed variables on $[0,1]$, the distributions of $1-r_i$ values are also uniform on $[0,1]$. This implies that each $R_k\ \forall k=0,\ldots,2^{N_{\rm eff}}$ follows a log-gamma distribution, mathematically given by $\frac{\ln^{N_{\rm eff}-1}\left(\frac{1}{p}\right)}{\Gamma(N_{\rm eff})}$, since they emerge as a product of $N_{\rm eff}$ independent standard uniform random variables. Based on these Eq. (S26) can be further simplified to

$$\rho_\gamma(p) = \frac{W-V^*}{W}\delta(p)+\eta\sum_{k=1}^{2^{N_{\rm eff}}}R_k = \frac{W-V^*}{W}\delta(p)+\eta\frac{\ln^{N_{\rm eff}-1}\left(\frac{1}{p}\right)}{\Gamma(N_{\rm eff})}\sum_{k=1}^{2^{N_{\rm eff}}}1 = \frac{W-V^*}{W}\delta(p)+\frac{V^*}{W}\cdot\frac{\ln^{N_{\rm eff}-1}\left(\frac{1}{p}\right)}{\Gamma(N_{\rm eff})}, \tag{S28}$$

where we have exploited the normalization constraint to specify the value of η.

Power-law density of states (power-law)

For the sake of technical controllability, let us define the following integral

$$I_{\text{Pow}}(A,m) = A\int_0^1 p^m \mathrm{d}p = \frac{A}{m+1}, \tag{S29}$$

based on which we can precisely evaluate various expressions later on. First, with the substitutions of $A = \frac{1}{W-1}$ and $m = -1 + \frac{1}{W-1}$ we obtain the normalization condition, whereas expected value requirement can be checked through $I_{\text{Pow}}\left(\frac{1}{W-1}, \frac{1}{W-1}\right) = \frac{(W-1)^{-1}}{(W-1)^{-1}+1} = \frac{1}{W}$.

Tsallis entropy can also be expressed as a function of the integral appearing in Eq. (S29), namely

$$S_q = W\frac{I_{\text{Pow}}\left(\frac{1}{W-1}, \frac{1}{W-1}\right) - I_{\text{Pow}}\left(\frac{1}{W-1}, q-1+\frac{1}{W-1}\right)}{q-1} = \frac{1}{q-1}\left(1 - \frac{W}{qW-q+1}\right), \tag{S30}$$

which reduces to BGS entropy if $q \to 1$, therefore

$$S_{\text{BGS}} = \lim_{q\to 1}\frac{1}{q-1}\left(1 - \frac{W}{qW-q+1}\right) = 1 - \frac{1}{W}, \tag{S31}$$

in a perfect accordance with Eq. (26).

An alternative formulation of power-law density of states is provided by the form of

$$\rho(p) = \frac{2}{W-4}\left(p^{-1+\frac{2}{W-2}} - 1\right), \tag{S32}$$

which has the nice property of $\rho(p=1) = 0$ but offerring the same type of scaling with W,

$$S_{\text{BGS}} = 3 - \frac{4}{W}. \tag{S33}$$

The fact that under power-law form of ρ both BGS and Tsallis entropies asymptotically converge to a finite value implies that these entropic forms can not display extensivity, not even in the limit of $W \to \infty$, therefore making it impossible to provide a thermodynamical description of the corresponding system.

Beta density of states (beta)

The Shannon entropy under Beta distribution of the configuration probabilities is given by the following integral

$$S_{\text{BGS}} = W\langle -p\ln p\rangle_\rho = W\int_0^1 \ln\left(\frac{1}{p}\right)\frac{p^a(1-p)^{b-1}}{B(a,b)}\mathrm{d}p = W\frac{a}{a+b}\left[\psi(a+b+1) - \psi(a+1)\right], \tag{S34}$$

where $\psi(x) = \frac{\mathrm{d}\ln\Gamma(x)}{\mathrm{d}x}$ denotes the digamma function. By imposing expected value constraint we obtain $\langle p\rangle_\rho = \frac{1}{W} = \frac{a}{a+b}$ based on which Eq. (S34) can be further simplified to

$$S_{\text{BGS}} = \psi(aW+1) - \psi(a+1). \tag{S35}$$

In addition to this, assuming scaling form for $a(W) \sim W^{\eta-1}$, we arrive to

$$S_{\text{BGS}} = \psi(W^\eta + 1) - \psi(W^{\eta-1}+1). \tag{S36}$$

Note that if $\eta > 0$ then $S_{\text{BGS}} \approx \psi(W^\eta) - \psi(1) \sim \eta\ln W$ asymptotically. As $\eta \to 0^+$ and $a^* \to \frac{1}{W-1} \sim W^{-1}$ a phase transition occurs whose presence can directly be derived from the recurrence relation of the Digamma function $\psi(x+1) = \psi(x) + \frac{1}{x}$. More precisely at the critical point a^*, where ρ decays as a power-law and extensivity is no longer accessible, Eq. (S36) displays the anomalous $S_{\text{BGS}} \sim 1 - \frac{1}{W}$ form. Above this transition point, beta distribution however, reduces to an exponential form under which anomalous scaling of the BGS entropy is obtained.

If first order statistics follows a beta distribution Tsallis entropy is given

$$S_q = \frac{1}{q-1}\left(1 - W\frac{\Gamma(a+q)\Gamma(aW)}{\Gamma(a)\Gamma(aW+q)}\right) = \frac{1}{q-1}\left(1 - W\frac{B(aW,q)}{B(a,q)}\right), \tag{S37}$$

where we have exploited the definiton of the beta function. Further algebraic manipulations for $a = 1$ where $\rho(p) \sim (1-p)^W$ and $B(W,q) \overset{W\to\infty}{\sim} \Gamma(q)W^{-q}$ yield to an asymptotic approximation of Tsallis entropies

$$S_q = \frac{1}{q-1}\left(1 - W(W+q)\Gamma(1+q)W^{-(1+q)}\right), \tag{S38}$$

scaling roughly as

$$S_q \approx \frac{1}{q-1}\left(1 - \Gamma(1+q)W^{1-q}\right). \tag{S39}$$

and giving H-T exponent $c = 1 - q$. For further details see Table1 and Table2.
